# The complete chloroplast genome sequence of *Zanthoxylum undulatifolium* Hemsl. (Rutaceae)

**DOI:** 10.1080/23802359.2022.2039084

**Published:** 2022-02-15

**Authors:** Chong Sun, Xia Liu, Houlin Zhou, Jing Liu, Xiaoying Li, Hailang Liu, Can He

**Affiliations:** a College of Horticulture and Gardening, Yangtze University, Hubei, China; b College of Landscape Architecture and Life Science, Chongqing University of Arts and Sciences, Chongqing, China; c Chongqing Wulipo National Nature Reserve Management Office, Chongqing, China

**Keywords:** *Zanthoxylum undulatifolium*, complete chloroplast genome, phylogenetic analysis

## Abstract

*Zanthoxylum undulatifolium* is an excellent economic tree species with important medical value. This study reports the first complete chloroplast genome sequence of *Z. undulatifolium*. Its whole chloroplast genome is 158,400 bp in length, including a large single-copy (LSC) region of 85,898 bp, a small single-copy (SSC) region of 17,610 bp, and two inverted repeat (IR) regions of 27,446 bp. The chloroplast genome contains a total of 132 genes, comprising 87 protein-coding genes, 37 tRNA genes, and eight rRNA genes. The overall GC content of the chloroplast genome is 38.46%, with the corresponding values in the LSC, SSC, and IR regions are 36.87%, 33.51%, and 42.55%, respectively. Phylogenetic analysis revealed the sister relationship between *Z. undulatifolium* and *Z. bungeanum*.

In 1895, W. Botting Hemsley, F. R. S. first published a description of *Zanthoxylum undulatifolium* Hemsl. as a new species (Hemsley 1895). *Z. undulatifolium* is a member of the genus *Zanthoxylum* L. in the family Rutaceae. It is a rare medicinal plant that narrowly distributed in the 1,600–2,300 m mountain forests or vegetation thicket areas of Southwest China (Editing Committee of Chinese Flora 1993). *Z. undulatifolium* is an excellent ecological tree species for soil and water conservation (Editing Committee of Chinese Flora 1993). No genomic information of *Z. undulatifolium* has been reported thus far. In this report, we present the first complete chloroplast genome sequence of *Z. undulatifolium* and construct its phylogenetic relationships with related species.

Samples of *Z. undulatifolium* was collected from Wushan County Goddess Peak, Chongqing, China (31.0461°N, 110.0281°E), and a voucher specimen was deposited at the Chongqing University of Arts and Sciences Herbarium (LYWS) under accession number CUAS-LY20180518 (Xia Liu, liuxiavip8@163.com). The genomic DNA was extracted from silica-dried leaf tissue using a modified CTAB method (Doyle and Doyle 1987). The DNA library was sequenced by Hefei Bio&Data Biotechnologies Inc. (Hefei, China) on the BGISEQ-500 platform with PE150 read lengths. The clean reads were used for the de novo assembly of the chloroplast genome using the SPAdes Assembler v3.9.0 (Bankevich et al. 2012). The annotation of the complete genome was performed using CpGAVAS (Liu et al. 2012) and GeSeq software (Michael et al. 2017). After a manual check and adjustment, the annotated chloroplast genome sequence of *Z. undulatifolium* was submitted to GenBank (MZ676708).

The chloroplast genome of *Z. undulatifolium* exhibited a typical angiosperm circular structure with a length of 158,400 bp and consisted of a large single-copy region (LSC: 85,898 bp), a small single-copy region (SSC: 17,610 bp), and two inverted repeat regions (IRs: 27,446 bp). The overall GC content of *Z. undulatifoliun* is 38.46% and the values in the LSC, SSC and IR regions are 36.87%, 33.51%, and 42.55%, respectively. The chloroplast genome encodes a total of 132 genes (87 protein-coding, 37 tRNA, and 8 rRNA genes), with 18 duplicated genes (7 protein-coding, 7 tRNA, and 4 rRNA genes). Nineteen genes contain two exons and four protein-coding genes (*ycf3*, *clpP*, and two *rps12*) contain three exons.

We performed a phylogenetic analysis based on the complete chloroplast genomes of 12 species and then constructed a phylogenetic tree to explore the phylogenetic relationships of *Z. undulatifolium* (Figure 1). The 12 complete chloroplast genome sequences were subjected to multile sequence alignment using MAFFT software (Katoh and Standley 2013). A maximum likelihood (ML) phylogenetic tree was built using the RAxML version 8 program (Alexandros 2014) with 1,000 bootstrap replicates. Phylogenetic analysis showed that *Z. paniculatum* and *Z. madagascariense* at the base of the phylogenetic tree are the oldest species among the selected *Zanthoxylum* species. *Z. undulatifolium* is most closely related to *Z. bungeanum*, and a sister to *Z. sp. NH018* and *Z. simulans*, with 100% bootstrap support values.

**Figure 1.**
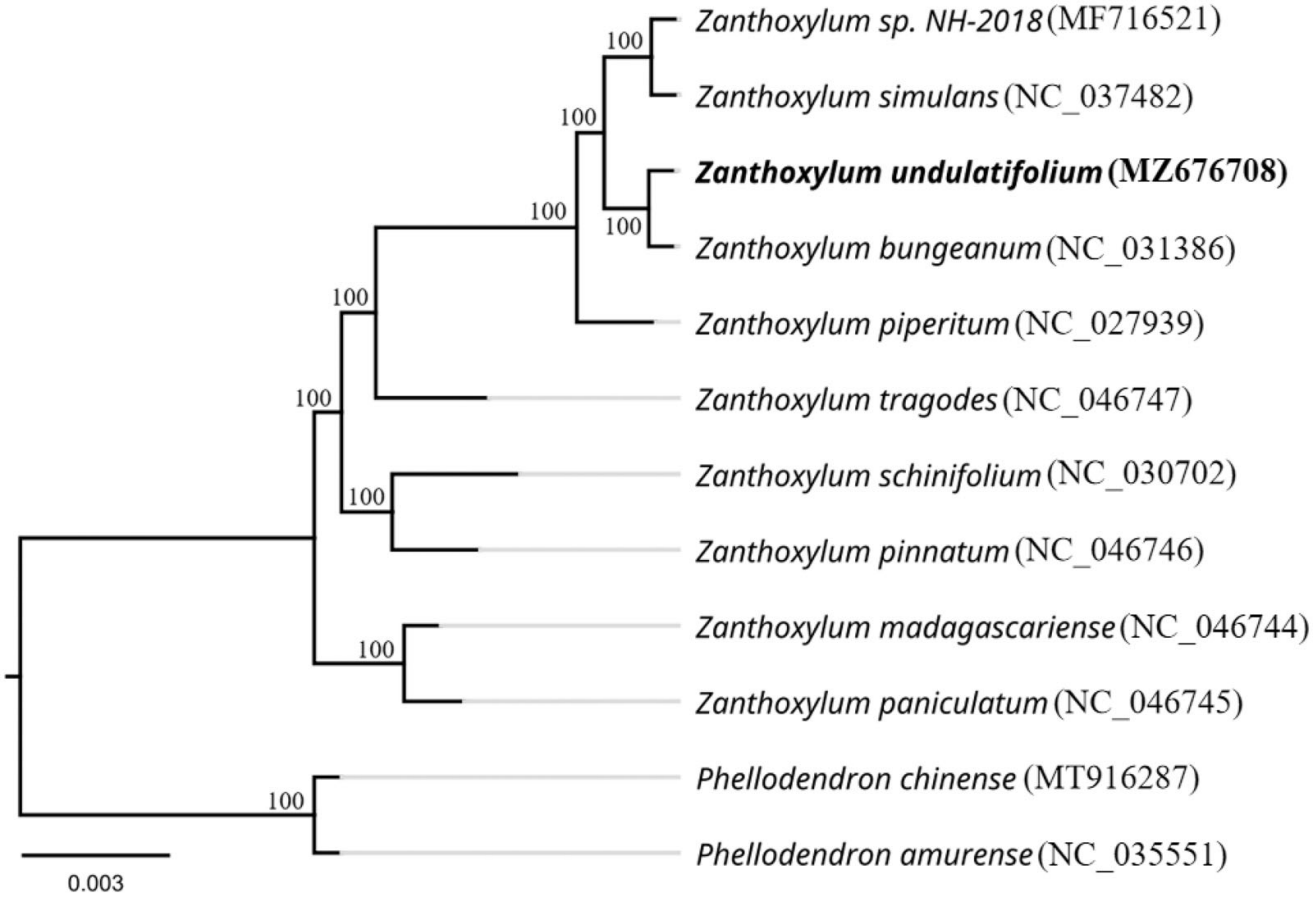
Maximum-likelihood phylogenetic tree of *Z. undulatifolium* and other related species based on the complete chloroplast genome sequences. The number on each node indicates the bootstrap support value.

A range of previous studies attempted to study the evolutionary relationships of *Zanthoxylum* species with molecular markers such as random amplified polymorphic DNA (Medhi et al. 2014), inter simple sequence repeat (Feng et al. 2015), isozyme (Li et al. 2004), simple sequence repeat (Kim et al. 2017), amplified fragment length polymorphism (Gupta and Mandi 2013), and chloroplast DNA markers (Appelhans et al. 2018). The limited number of polymorphic loci produced by these low-resolution markers hinders phylogenetic research on *Zanthoxylum* species. Therefore, (i) we should obtain more samples of *Zanthoxylum* from China, North America and Japan, and (ii) we should study the evolutionary relationship based on the complete chloroplast genome of these *Zanthoxylum* species to examine the evolution relationships of *Zanthoxylum* species. In this paper, the complete chloroplast genome sequence of a representative *Zanthoxylum* species provides important insights into the evolution of *Zanthoxylum* in eastern Asia.

## Data Availability

The genome sequence data that support the findings of this study are openly available in GenBank of NCBI at (https://www.ncbi.nlm.nih.gov/) under the accession no. MZ676708. The associated BioProject, SRA, and Bio-Sample numbers are PRJNA680256, SRR17163936, and SAMN23766596, respectively.
